# Epidemiological study of Ectopic Pregnancy at Sulaimani Maternity Teaching Hospital, Iraq: A cross-sectional study

**DOI:** 10.1097/MD.0000000000049024

**Published:** 2026-05-29

**Authors:** Naz Azad Abdullah, Rozhan Khalid Hassan, Asia Kawa Muhammed, Israa Musa Muhammed

**Affiliations:** a Branch of Clinical Sciences, College of Medicine, University of Sulaimani, 0047 Sulaymaniyah, Iraq; b Department of Gynecology and Obstetrics, Sulaimani Teaching Hospital, Sulaimani Directorate of Health, 0046 Sulaymaniyah, Iraq.

**Keywords:** Ectopic pregnancy, incidence, infertility, pelvic inflammatory disease, risk factors

## Abstract

Ectopic pregnancy (EP) remains a major cause of early pregnancy-related morbidity and mortality. It can significantly impact future fertility and is associated with various risk factors. To investigate the incidence, clinical profile, and risk factors of EP among admitted women to the tertiary hospital. A prospective, cross-sectional study was conducted on 393 women with early pregnancy at the Maternity Teaching Hospital, Sulaymaniyah, Iraq from January 2024 to April 2025. Collected data included patients’ demographic characteristics, clinical data, and risk factors for EP, using a structured questionnaire. Then, diagnosis of EP was confirmed by serum β-hCG test and transvaginal ultrasonography. Management approaches were applied (manual, expectant, or surgical). Among studied women (n = 393) with early pregnancy, 79 (20.1%) were diagnosed with EP. The mean age of EP patients was 31 ± 4.47 years and mostly aged 30 to 39 years (44.3%), from urban area (68.4%), housewives (86.1%), gave birth to 2–4 children (62%), had 1–2 pregnancies (%). Frequent symptoms included lower abdominal pain (82.3%) and vaginal bleeding (67.1%) (*P* = .047). Major observed risk factors were previous C/S (54.4%), pelvic inflammatory disease (48.1%), and prior EP (11.4%) (*P* < .001). Tubal pregnancies constituted 82.3%, followed by C/S scar (15.2%), and rare cervical and ovarian sites (1.3%) (*P* < .001). Management was mainly surgical (50.6%), then expectant (25.3%) approach, and medical treatments (24.1%) (*P* = .049). The incidence of EP was higher than global estimates, likely due to the study population characteristics. However, this single-center study is not a representative of the regional epidemiology of ectopic pregnancy.

## 1. Introduction

Ectopic pregnancy (EP) refers to the implantation of a gestational sac outside the uterine cavity, and its incidence is about 1% in Western women of reproductive age.^[1]^ Due to its potential for rupture and hemorrhage, it is considered a high-risk condition and the leading cause of 9 to 14% maternal mortality in the first trimester.^[2]^ Women with EP may experience severe morbidity, such as an increased risk of recurrence and future infertility; thus its early detection is critical for conservative treatment and improving outcomes.^[3]^ The classic presentation of an EP is the triad of abdominal or pelvic pain, vaginal bleeding, and the presence of an adnexal mass. About 95% of EP occur in the fallopian tubes, making tubal implantation the most common form of EP, especially in the ampulla region (70%), while less common sites include the ovaries (3%), cervix, myometrium, cesarean section (C/S) scar, and peritoneal cavity (<1% each).^[4,5]^ Approximately half of all EP occur in women without identifiable risk factors.^[6]^ Factors associated with an increased risk of EP include a history of prior EP, that occurs in 10% of women after 1 previous EP and > 25% after ≥ 2.^[4]^ Other risk factors include advanced maternal age (>35 years), cigarette smoking, history of pelvic infection or pelvic inflammatory disease (PID), increasing use of assisted reproductive technology (ART),^[7]^ prior fallopian tube surgery, previous pelvic or abdominal surgery, certain sexually transmitted infections, and endometriosis.^[7]^ It was found that individuals with intrauterine contraceptive devices (IUCDs) are at lower risk of EP than those who do not use it; however, about 53% of pregnancies that occur with an IUD in situ are ectopic.^[2]^

Current diagnostic methods for EP rely on the correlation between serum beta human chorionic gonadotropin hormone (β-hCG) levels and image findings, in which transvaginal ultrasound has been shown to be more accurate and sensitive compared to transabdominal ultrasound. Expectant management is the most conservative approach and is appropriate for patients with declining or plateaued β-hCG levels.^[2]^ In cases with the initial β-hCG level of < 200 mIU/mL; about 88% of EPs resolve spontaneously. Although rarely employed, expectant management may be considered in carefully selected asymptomatic patients with low and decreasing β-hCG who are willing to accept the risk of tubal rupture or hemorrhage.^[8]^ Methotrexate, a folic acid antagonist, is an effective pharmacologic treatment for early, unruptured EPs, especially in cases with lower baseline β-hCG.^[9]^ Surgical intervention (salpingectomy or salpingostomy) remains a cornerstone in the management of EP, in which ultrasound detection of an extra-uterine embryo with fetal cardiac activity indicates an urgent need for surgical treatment.^[10]^ For hemodynamically stable patients who desire future fertility, a combination of salpingostomy and methotrexate is highly effective with lower failure rate than either treatment alone and minimal impact on quality of life.^[11]^ Up-to-date, there is no detailed publish data in Iraq, especially in this locality concerning the epidemiology of EP, although some studies were performed on incidence, risk factors and treatment outcomes of EP in different cities of Iraq.^[12–14]^ Therefore, this study aimed to find the incidence, clinical profile, and observed risk factors of EP among women.

## 2. Patients and methods

### 2.1. Study design and setting

This prospective, cross-sectional study was conducted on 393 pregnant women admitted to the Sulaimani Maternity Teaching Hospital, Sulaimaniyah, Iraq, from January 2024 to April 2025.

### 2.2. Inclusion criteria

Women that diagnosed with an EP, regardless of its type.

### 2.3. Exclusion criteria

Women with uncertain diagnostic confirmation.

### 2.4. Study protocol

A self-developed, validated questionnaire was used to collect patients’ data, such as demographic characteristics (age, residency, occupation, and blood group), obstetrical and gynecological history (parity, gravidity, history of miscarriage, and presenting symptoms), and risk factors for EP (contraceptive use, ART use, history of PID, previous C/S, previous EP, previous pelvic/abdominal surgery, smoking, and site of EP). Then, diagnosis of EP was confirmed by serum β-hCG test and transvaginal ultrasonography. Management approaches were applied, including either methotrexate injection, expectant management, or surgical intervention.

### 2.5. Statistical analysis

The IBM Statistical Package for Social Science (version 25, Chicago, USA) was used to analyze the data. Descriptive statistics were used to summarize the data, including frequencies, percentages.

## 3. Results

During this study period over 4 months, a total of 393 early pregnant cases were admitted to the Sulaimani Maternity Teaching Hospital. Among these, 79 (20.1%) cases were diagnosed as EP. The mean maternal age of patients with EP was 31 ± 4.47 years, with ages ranging from 18 to 45 years. Majority were aged 30–39 years (44.3%), 68.4% resided in urban areas, and were housewives (86.1%). The residency and occupation were significantly associated with the occurrence of PE (*P* ≤ .05) (Table 1). The mean gravidity (*P* ≤ .05), parity (*P* ≤ .05), and miscarriage (*P* ≥ .05) of patients with EP were 3.58 ± 2.054 (ranged from 1–4), 1.63 ± 1.379 (ranged from 1–6), and 0.949 ± 1.298 (ranged from 0–3), respectively. Most EP patients were significantly presented with lower abdominal pain (82.3%) and vaginal bleeding (67.1%) (*P* ≤ .05) (Table 2). Regarding the risk factors for EP among patients, 43 cases (54.4%) had a history of previous CS, along with 9 cases (11.4%) with a history of previous EP (*P* < .001). Also, 6 cases (7.6%) had a history of previous IUCD use, while 8 cases (10.1%) were current IUCD users (*P* < .001). Only 1 EP occurred in a patient underwent ART (1.3%). Simultaneously, 38 patients had a history of PID as a risk factor for EP (48.1%). A history of previous pelvic and/or abdominal surgery was reported in 13 cases (16.5%). None of the patients were smokers. The majority of EPs were tubal in location (n = 65, 82.3%), while scar EP was found in 12 (15.2%) cases, and there was 1 cervical and 1 ovarian in location (1.3% each) (*P* < .001) (Table 3). Regarding management of the EP cases, 40 (50.6%) of them were managed surgically, while 20 (25.3%) were managed expectantly and 19 (24.1%) received medical treatment (*P* = .049) (Table 4).

**Table 1:** Demographic characteristics of the enrolled patients.

Variable		Frequency	Percentage	*P*-value
Age (Years)	18–20	3	3.8	.075
	21–29	31	39.2	
	30–39	35	44.3	
	≥40	10	12.7	
Residency	Rural	25	31.6	.04*
	Urban	54	68.4	
Occupation	House-wife	68	86.1	.034*
	Student	1	1.3	
	Self employed	6	7.6	
	Public servant	4	5.1	
**Total**		**79**	**100**	

* Significant difference using Chi-square test.

**Table 2:** Obstetrical profile and presenting symptoms of patients with ectopic pregnancy.

Variable		Frequency	Percentage	*P*-value
Gravidity	Primigravida	11	13.9	.045*
	2–4	49	62.0	
	≥5	19	24.1	
Parity	Nulliparous	21	26.6	.038*
	1–2	38	48.1	
	3–4	18	22.8	
	≥5	2	2.5	
Miscarriage	0	34	43.3	.65
	1	32	40.5	
	≥2	12	15.2	
Presenting symptom	Pain	65	82.3	.047*
	Vaginal bleeding	53	67.1	
	Vomiting	6	7.6	
	Fainting	1	1.3	
**Total**		**79**	**100**	

IUCD = Intrauterine contraceptive device, PID = Pelvic inflammatory diseases.

* Significant difference using Chi-square test.

**Table 3:** Observed gynecological risk factors for ectopic pregnancy among the patients.

Variable		Frequency	Percentage	*P*-value
Previous cesarean section	No	36	45.6	.55
	Yes	43	54.4	
Previous ectopic pregnancy	No	70	88.6	<.001
	Yes	9	11.4	
Previous IUCD user	No	73	92.4	<.001
	Yes	6	7.6	
Current IUCD user	No	71	89.9	<.001
	Yes	8	10.1	
Oral contraceptive pills user	No	75	94.9	<0.001
	Yes	4	5.1	
Assisted reproductive technology	No	78	98.7	<0.001
	Yes	1	1.3	
History of PID	No	41	51.9	<0.001
	Yes	38	48.1	
Previous pelvic/abdominal surgery	No	66	83.5	<0.001
	Yes	13	16.5	
Smoking	No	79	100.0	<0.001
	Yes	0	0.0	
Sites of ectopic pregnancy	Tubal	65	82.3	<0.001
	Scar ectopic	12	15.2	
	Ovarian	1	1.3	
	Cervical	1	1.3	
**Total**		**79**	**100**	

* Significant difference.

** Highly significant difference, using Chi-square test.

**Table 4:** Treatment modality for ectopic pregnant patients.

Management	Frequency	Percentage	*P*-value
Medical	19	24.1	.049*
Surgical	40	50.6	
Expectant	20	25.3	
**Total**	**79**	**100**	

* Significant difference using Chi-square test.

## 4. Discussion

An EP is a life-threatening condition that requires emergency treatment as the fallopian tube is not make to hold the growing embryo.^[15]^ Thus, this study aimed to assess the incidence of this condition among pregnant women who were admitted to Sulaimani Maternity Teaching Hospital over 4 months’ period with its clinical profile, risk factors and potential management patterns. Consequently, it was found that EP accounted for 20.1% of admissions with early pregnancy. This proportion is higher than that reported by Joshi, 2025 (2.1%),^[16]^ Gaskins et al. (1.0%),^[17]^ Ranji et al., 2018 (2.81%),^[18]^ and Mahmood, 2019 (0.132%).^[19]^ These variations might be related to sample size and the selective nature of sampling, in which this study included only women presenting with early pregnancy during 4 months at a single tertiary hospital, rather than representing true population incidence.

Additionally, in this study the majority of EP occurred in patients aged 30 to 39 years (44.3%), with a mean age of 31 years. This finding is comparable to the case–control study by Moini et al.,^[20]^ which observed a higher frequency of EP among older women, that possibly related to age-related changes in tubal function. In contrast, Godria et al.,^[21]^ reported most EP cases among women aged 20 to 30 years, while Ranji et al.,^[18]^ reported 26 to 30 years. These outcomes may reflect different fertility patterns in their study populations.

Moreover, in this study, most patients were multigravida, with 62% having 2 to 4 previous pregnancies and 24.1% having ≥ 5, while only 13.9% were primigravida. These findings are lower than Kumari et al., which reported 76.4% of EP cases in multigravida women,^[22]^ but higher than Malik et al., who found 56.2% of EP women were multigravida.^[23]^ Another study stated a significant correlation between an EP and having an induced abortion of > 2 in the past.^[24]^ This contradicts our research, which shows that 15.2% of patients had ≥ 2 abortions, with 43.3% of patients never had an abortion. Furthermore, this study found that 48.1% of patients had a history of PID, similar to the observations of Mahajan et al.^[25]^ Similarly, Muzaffar et al mentioned that PID, particularly Tuberculosis and Chlamydia are major contributing factors for EP.^[26]^ These might be owed to that an infection-induced inflammation causes scarring in the fallopian tubes that blocks the fertilized egg from reaching the uterus. Thus, PID should be treated with the early medical consultation when symptoms occur and recommending to use a barrier method for contraception to reduce the risk of PID associated with sexually transmitted pathogens.^[27]^ On contrary, Malik et al found PID in only 1.3% of EP patients.^[23]^

Because of the rising number of C/S, the scar area has been identified as an additional site for EP implantation. This condition is rare but serious that known as cesarean scar pregnancy. Thus, in the current study, 54.4% of women with at least 1 prior C/S had EP, which is consistent with that of O’Neill et al.^[28]^ On contrary, Bowman et al.^[29]^ stated that women who had 1 prior C/S were not at increased risk for subsequent EP in relation to those with no prior C/S. However, women with 2/2, 2/3, or 3/3 prior C/S had increased risk for subsequent EP.^[29]^ Although having previous EPs are a well-known risk factor for future EP, as reported by Marlina et al with recurrence risks of 10 to 25% depending on the number of prior EP.^[30]^ However, in this study, only 9 patients (11.4%) had a history of previous EP; thus, it is considered as a weak risk factor. This finding is similar to that of by Malik et al. (9.2%).^[23]^

Generally, using contraceptives reduces the overall risk of EP by preventing pregnancy completely. However, if a pregnancy occurs while using specific methods; particularly progestin pills, implants, or IUCDs; the proportion of those rare, breakthrough pregnancies that are ectopic is higher. However, previous use does not cause future risk.^[31]^ In this study, current use of IUCDs was identified in only 10.1% of EP patients, which is consistent with the findings of Schultheis et al.,^[19]^ who reported a decreased risk of EP among IUCD users. However, prior use of oral contraceptive pills in this study was not a significant feature, as only 4 patients had a history of such use, which also aligns with another studies.^[20,32]^

It is well known that ART leads to a higher incidence of EP (2 to 2.5-fold) compared to spontaneous conceptions,^[33]^ but the underlying etiology remains unclear. In this study, only 1 EP pregnant women (1.3%) had ever used ART; thus, ART is not considered as a strong risk factor for EP. However, some studies declared that tubal infertility, the number of embryos transferred, length of embryo culture and some metabolic pathways and proteins are significantly associated with the incidence of EP.^[34]^

Prior pelvic or abdominal surgery considered a significant risk factor for EP, due to adhesions, scarring, or anatomical distortion of the fallopian tubes, which interferes with the fertilized egg journey to the uterus. Similarly in to this study, 16.5% of patients had a history of prior pelvic or abdominal surgery, which is higher than that of Mahajan et al., who found 10.3% of patients had a prior pelvic surgery (tubal ligation and other tubal surgery).^[25]^

Based on the outcomes of Flanagan et al.,^[35]^ cigarette smoking is a major risk factor for tubal EP. This might be related to the cigarette smoke component; Cotinine, which is the active component in nicotine and has been shown to cause changes to embryo transport, embryo-tubal interactions and the tubal microenvironment.^[36]^ However, none of the patients in this study were smokers. Obviously, tubal EP is the most common form of EP and we found 82.3% of EPs were tubal, which is slightly lower than a previous report (95%).^[5]^ In contrast, we revealed a lower proportion of EPs located at a previous C/S scar site (15.2%), while 54.4% of the women had a history of C/S. This may reflect an increasing rate of C/S in our population. The choice of EP treatment modality depends on clinical presentation and resource availability. Consequently, all EP patients in this study were treated, of which surgical management was predominant (50.6%). Whereas Malik et al stated that surgical management was required in 61.5% of cases, while 11.8% were managed medically.^[23]^

However, this study has several limitations, as it is a prospective descriptive hospital-based cross-sectional study; causal relationships between observed risk factors and EP could not be established. Because only admitted cases at a single tertiary maternity teaching hospital were included, the findings may not represent the broader population and selection bias is possible.

## 5. Conclusions

EP remains a significant source of reproductive health morbidity, so knowing their rate and risk factors helps Gynecologists to manage women for their future fertility and even psychological support. Therefore, targeted health education campaigns should be conducted to enlighten these women, well educate them about the risk factors and outcomes, and aware them of the increased risk of such abnormal pregnancies with the rising rate of C/S. Also, awareness of key risk factors, such as prior surgeries, infections, and reproductive history are crucial for early diagnosis and prevention. Finally, we are recommending an improved accessibility to family planning, early screening, and psychological support for women of reproductive age to ensure early diagnosis and reduce complications.

## Acknowledgments

We would like to express special thanks to the College of Medicine, University of Sulaimani, and healthcare staff of Sulaimani Maternity Teaching Hospital, Sulaymaniyah, Iraq for their support and cooperation during this study.

## Author contributions

**Conceptualization:** Naz Azad Abdullah.

**Methodology:** Naz Azad Abdullah, Rozhan Khalid Hassan, Asia Kawa Muhammed, Israa Musa Muhammed.

**Supervision:** Naz Azad Abdullah.

**Writing – review & editing:** Naz Azad Abdullah.

**Data curation:** Rozhan Khalid Hassan, Israa Musa Muhammed.

**Formal analysis:** Rozhan Khalid Hassan, Israa Musa Muhammed.

**Investigation:** Rozhan Khalid Hassan, Asia Kawa Muhammed.

**Writing – original draft:** Rozhan Khalid Hassan, Asia Kawa Muhammed, Israa Musa Muhammed.

**Project administration:** Asia Kawa Muhammed.

**Resources:** Asia Kawa Muhammed.
